# Beyond One-Size-Fits-All: Individually Tailored Dietary Interventions in Modern Obesity Care

**DOI:** 10.3390/nu18162691

**Published:** 2026-08-18

**Authors:** Anamaria Cozma-Petruț, Maria Gherghel, Roxana Banc, Ioana Badiu Tișa, Oana Mîrza, Teodora Emilia Coldea, Elena Mudura, Doina Miere, Lorena Filip

**Affiliations:** 1 Department of Bromatology, Hygiene, Nutrition, Faculty of Pharmacy, “Iuliu Hațieganu” University of Medicine and Pharmacy, 6 Pasteur Street, 400349 Cluj-Napoca, Romania; anamaria.cozma@umfcluj.ro (A.C.-P.); gherghel.maria@elearn.umfcluj.ro (M.G.); oana.stanciu@umfcluj.ro (O.M.); dmiere@umfcluj.ro (D.M.); lfilip@umfcluj.ro (L.F.); 2 Department of Nursing, Faculty of Nursing and Health Sciences, “Iuliu Hațieganu” University of Medicine and Pharmacy, 2-4 Câmpeni Street, 400217 Cluj-Napoca, Romania; ioana.badiu@umfcluj.ro; 3 Laboratory of Toxicology, Institute of Forensic Medicine Cluj-Napoca, 3-5 Clinicilor Street, 400006 Cluj-Napoca, Romania; 4 Department of Food Science, Faculty of Food Science and Technology, University of Agricultural Sciences and Veterinary Medicine Cluj-Napoca, 400372 Cluj-Napoca, Romania; teodora.coldea@usamvcluj.ro; 5 Department of Food Engineering, Faculty of Food Science and Technology, University of Agricultural Sciences and Veterinary Medicine Cluj-Napoca, 400372 Cluj-Napoca, Romania; elena.mudura@usamvcluj.ro; 6 Academy of Romanian Scientists (AOSR), 3 Ilfov Street, 050044 Bucharest, Romania

**Keywords:** obesity, individualized dietary interventions, precision nutrition, obesity-related genes, microbiota modulation, metabotypes, lipidomics, incretin therapy, artificial intelligence

## Abstract

Obesity is a complex, multifactorial chronic disease characterized by excessive and/or aberrant adiposity that requires interventions beyond conventional calorie-restriction models. Current clinical guidelines converge on behavioral modification, encompassing nutritional therapy, physical activity, stress reduction, and sleep improvement, as the cornerstone of obesity management, with psychological therapy, pharmacotherapy, and bariatric procedures as adjunctive or escalating options. While traditional “one-size-fits-all” dietary approaches frequently yield suboptimal long-term outcomes, precision nutrition has emerged as a promising strategy for individualized obesity care. This review critically appraises the evidence underlying each pillar of precision nutrition: genetic profiling shows clear utility in monogenic obesity but variable benefit when diet is matched to common polygenic variants; microbiome-targeted studies reveal that the *Bacillota* to *Bacteroidota* ratio shows inconsistent associations with obesity across studies, with genus-level and functional alterations offering more reproducible targets; and metabolomic profiling of postprandial glycemic responses has progressed from foundational predictive algorithms to large-scale clinical validation. It also highlights chrono-nutrition, the alignment of food timing with individual chronotype, as an emerging pillar of personalization. Beyond dietary composition, the review addresses how personalized nutrition can mitigate the adverse effects of incretin-based pharmacotherapy and discusses how artificial intelligence can integrate multi-omics and behavioral data into real-time dietary guidance. Future clinical implementation will require overcoming challenges related to data integration, predictive accuracy, and accessibility, paving the way for more effective, evidence-based obesity management.

## 1. Introduction

Obesity represents a major global health issue characterized by excessive and/or aberrant adiposity that significantly impairs physical health, quality of life, and life expectancy. The rising prevalence of obesity and its associated non-communicable diseases (NCDs), such as type 2 diabetes and cardiovascular disease, emphasize the urgent need for effective therapeutic strategies [1,2]. Current management guidelines converge on behavioral modification, encompassing nutritional therapy, physical activity, stress reduction, and sleep improvement, as the foundational cornerstone of obesity care, with psychological therapy, pharmacotherapy, and metabolic or bariatric procedures (surgical and endoscopic) considered as adjunctive or escalating options for patients who do not achieve sufficient benefit from lifestyle intervention alone [3].

Historically, weight management has relied heavily on the “calories in, calories out” framework and standardized dietary interventions. However, this conventional “one-size-fits-all” approach often fails to achieve sustainable weight loss due to substantial interindividual differences in metabolic responses, driven by variability in genetic predisposition, body composition, insulin sensitivity, hormonal fluctuations, and lifestyle habits (e.g., exercise, sleep, stress) [4]. In recent years, the paradigm of obesity care has shifted towards precision nutrition, an approach that tailors dietary recommendations to an individual’s unique biological and lifestyle characteristics. By capitalizing on advances in omics technologies, including genomics, metabolomics, and microbiomics, precision nutrition seeks to identify the specific drivers of metabolic dysfunction in each patient [5]. Concurrently, the advent of highly effective incretin-based pharmacotherapies, such as glucagon-like peptide-1 (GLP-1) receptor agonists, has revolutionized clinical obesity management. Nevertheless, these pharmacological treatments present new nutritional challenges, requiring personalized dietary strategies to manage gastrointestinal adverse effects, prevent nutritional deficiencies, and preserve lean body mass [6].

Furthermore, the integration of artificial intelligence (AI) and machine learning (ML) offers unprecedented opportunities to analyze complex multi-omics data, enabling dynamic, individually tailored nutritional interventions. Recent progress in AI for precision nutrition has expanded across domains such as dietary assessment, phenotyping, risk prediction, and the generation of personalized dietary recommendations. By using ML algorithms to decipher intricate genomic and metabolic data, AI has the potential to translate complex biological information into actionable, real-time nutritional guidance, ultimately paving the way for more sustainable and effective obesity management [4].

This review aims to comprehensively evaluate the limitations of traditional dietary models and delineate the core pillars of precision nutrition, highlighting its integration with modern pharmacotherapy and the transformative potential of AI in advancing individualized obesity care.

## 2. Methodology

The present manuscript is a non-systematic, state-of-the-art narrative literature review synthesizing the current knowledge on the individualized management of obesity. As a narrative review, this work does not follow a PRISMA-based protocol, was not registered in advance, and does not report a quantitative synthesis (e.g., meta-analysis) of the retrieved evidence. Instead, it provides a critical, integrative overview of the field intended to contextualize and interpret the existing literature. A comprehensive literature search was conducted in PubMed, Scopus, and Web of Science, covering publications from January 2017 to June 2026.

A wide range of keywords and Medical Subject Headings (MeSH) terms were utilized to ensure thorough coverage, including but not limited to: obesity, precision nutrition, personalized nutrition, individualized dietary interventions, genetics, nutrigenetics, genome-wide association studies (GWAS), gut microbiome, probiotics, prebiotics, fecal microbiota transplantation, metabolomics, lipidomics, incretin-based pharmacotherapies, GLP-1 receptor agonists, dual GIP/GLP-1 receptor agonists, weight management, interindividual variability, caloric restriction, artificial intelligence, and machine learning. Boolean operators (AND, OR, NOT) were systematically applied to refine and optimize the search strategy across all databases. The inclusion criteria were: (1) peer-reviewed original research articles, clinical trials, systematic reviews, meta-analyses, and narrative reviews published in English between January 2017 and June 2026; (2) articles addressing the pathophysiology of obesity and the mechanisms underlying interindividual variability in responses to diet; (3) publications exploring omics-based approaches in the context of personalized obesity management; (4) studies examining the nutritional implications and dietary optimization strategies associated with incretin-based pharmacotherapies; and (5) publications investigating the application of artificial intelligence and machine learning in precision nutrition and obesity care.

Scientific articles were first analyzed based on their titles and abstracts to establish relevance to the review’s scope. Subsequently, full-text evaluations were performed for all articles meeting the inclusion criteria. Furthermore, epidemiological data were obtained from the official websites of relevant international health organizations and regulatory agencies. The findings were critically synthesized to present an integrated, up-to-date perspective on the individually tailored dietary interventions in modern obesity care.

**Figure 1:**
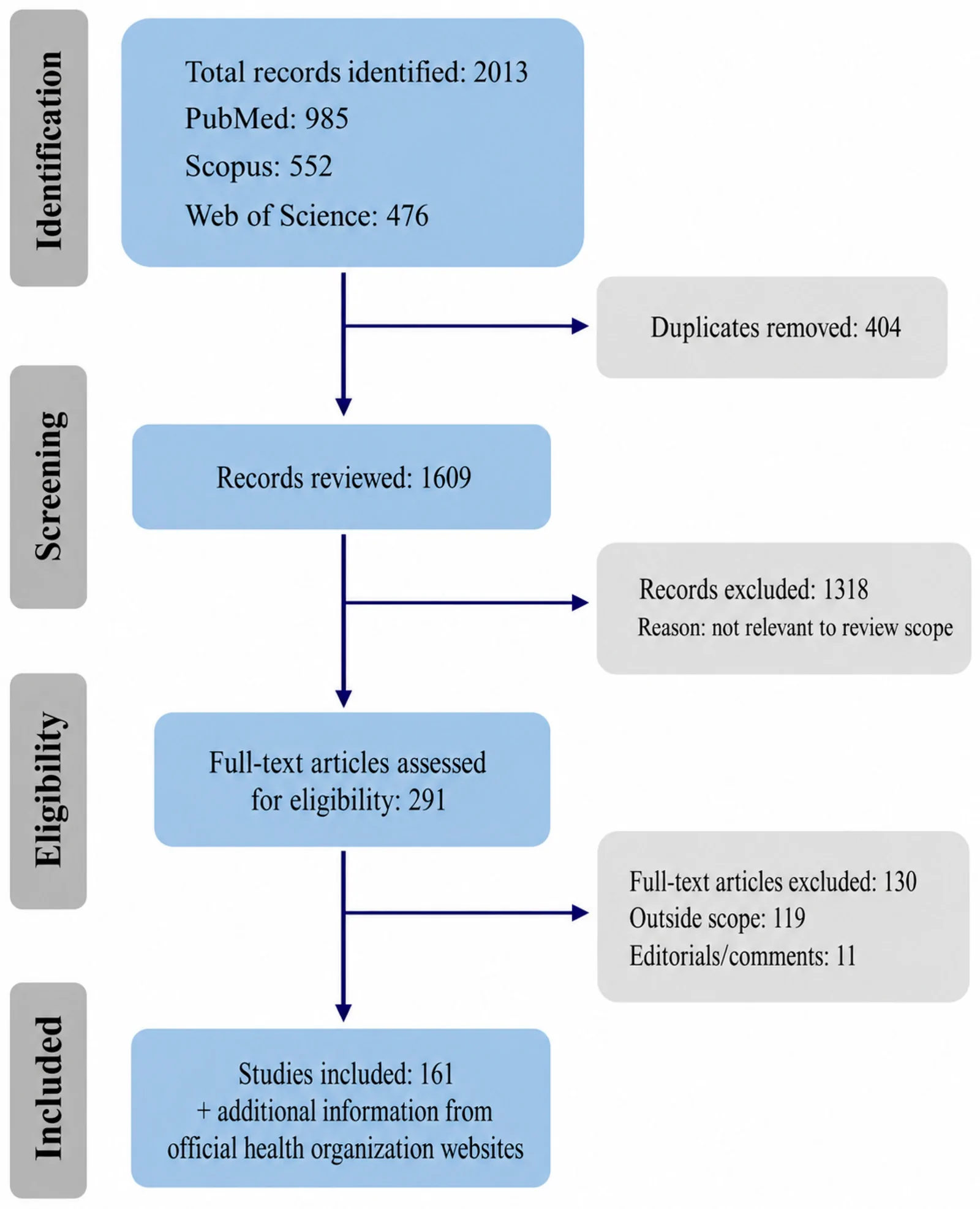
PRISMA-inspired flow diagram for article selection.

To improve the transparency and reproducibility of the search and selection process, a PRISMA-inspired flow diagram summarizing the number of records identified, duplicates removed, records screened, full-text articles assessed, and studies ultimately included is presented in Figure 1. It should be noted that, in agreement with the non-systematic nature of this narrative review, the selection process was not guided by a pre-registered protocol, and articles were included based on thematic alignment as deemed by the authors rather than a standardized, reproducible screening algorithm applied independently by multiple reviewers.

## 3. The Global Burden of Obesity

Obesity has become a global epidemic, standing out as one of the major health crises of the 21st century [7]. According to the World Health Organization (WHO), between 1990 and 2022, adult obesity rates more than doubled, while in children and adolescents, the prevalence has quadrupled, outpacing the rate of increase seen in adults. Formerly seen as a challenge for high-income countries alone, obesity now extends across all regions of the world, affecting people of every socioeconomic background. In 2022, approximately one in eight people worldwide lived with obesity, accounting for nearly 880 million adults and about 159 million children and adolescents [8,9].

In the United States specifically, obesity prevalence rose dramatically, reaching 40.3% among adults between 2021 and 2023 [10,11]. A similar pattern is observed in Europe, where around 59% of adults live with overweight or obesity, representing a major risk factor for NCDs [12]. These trends are expected to worsen globally: projections from the World Obesity Atlas suggest that by 2030, the number of adults living with obesity will exceed 1.13 billion, and by 2040, childhood obesity will surpass childhood underweight globally [13,14].

Beyond these projections, it is important to acknowledge that obesity already represents a serious threat to public health, substantially increasing the risk of type 2 diabetes, cardiovascular disease, dyslipidemia, hypertension, at least 13 distinct cancers, obstructive sleep apnea, and osteoarthritis [15]. In the case of type 2 diabetes, the American Diabetes Association (ADA) emphasizes that obesity is a primary driver of the disease, accounting for up to 53% of new cases every year in the United States [16]. Furthermore, obesity acts as a risk factor for infection-related hospitalizations and death across a wide range of pathogens, populations, and clinical backgrounds [17]. The magnitude of this burden is highlighted by epidemiological data: in 2019, a high body mass index (BMI) was responsible for approximately 5 million deaths globally. In the WHO European Region alone, overweight and obesity accounted for more than 1.2 million deaths annually, making them the fourth leading NCD risk factor in the region, after hypertension, diet quality, and tobacco use [7,12,15].

Obesity reduces life expectancy and significantly diminishes quality of life. The financial impact is staggering, with projections indicating that the worldwide economic burden of overweight and obesity could rise to US$4.32 trillion per year by 2035. This emphasizes the urgent need for effective, sustainable, and individualized public health and clinical interventions [7,18].

## 4. The Current Definitions of Obesity

Obesity is recognized as a chronic, complex condition characterized by an excessive and/or aberrant accumulation of fat, which can detrimentally impact both physical health and overall quality of life [8]. Traditionally, the main approach for identifying overweight and obesity has been the BMI, determined by dividing a person’s weight in kilograms by the square of their height in meters (kg/m^2^) [8,19,20,21,22]. Adults with a BMI of 25–29.9 kg/m^2^ are classified as overweight or pre-obese, while a BMI of 30 kg/m^2^ or higher indicates obesity [19], which is further divided into three classes based on severity [20]. Table 1 summarizes the BMI categories for adults and their corresponding classifications.

Nevertheless, relying exclusively on BMI to diagnose obesity is problematic because it does not differentiate between fat, muscle, and bone mass, nor does it indicate the specific type or location of fat in the body [21,23,24]. To address this, major health organizations recommend measuring waist circumference to identify visceral fat accumulation, a simple and inexpensive method that strongly predicts metabolic risk. Moreover, bioelectrical impedance analysis (BIA) represents an accessible and non-invasive alternative for estimating body fat percentage and lean mass, offering a practical intermediate option between simple anthropometric measures and advanced imaging techniques. For a more precise quantification of body composition, dual-energy X-ray absorptiometry (DEXA) can be utilized, though its routine clinical use is limited by cost and availability [8,21,25].

Recent advancements in the definition of obesity reflect a shift towards evaluating functional health rather than just body size. In 2024, the European Association for the Study of Obesity (EASO) proposed that an individual is considered obese if they have a BMI of 30 or higher, or a BMI of 25 or more, along with a waist-to-height ratio ≥ 0.5, together with obesity-associated health, functional, or psychological conditions [3].

Furthermore, the Lancet Commission on Clinical Obesity recently proposed a framework distinguishing between “clinical obesity” (excess adiposity causing measurable impairment of organ function or substantially restricting daily functioning, warranting active medical treatment) and “preclinical obesity” (excess body fat without current functional consequences but with elevated risk of progression to clinical obesity and associated NCDs). Classification requires confirmation of excess adiposity using BMI combined with at least one body size measurement (waist circumference, waist-to-hip ratio, or waist-to-height ratio); at least two body size measurements without BMI; or direct body fat quantification by DEXA. A BMI above 40 kg/m^2^ is considered sufficient on its own. This multi-criteria approach addresses the limitations of BMI and better reflects the actual health trajectory of the patient [23].

The clinical heterogeneity of obesity is further illustrated by the concepts of metabolically healthy obesity (MHO) and metabolically unhealthy obesity (MUO). No universally accepted definition of MHO has been established, with most studies classifying individuals as metabolically healthy based on the absence of, or a limited number of, metabolic syndrome components, though criteria may also incorporate markers of insulin resistance or systemic inflammation. MHO is generally characterized by preserved insulin sensitivity and predominantly subcutaneous rather than visceral fat accumulation. Reported prevalence estimates vary considerably across studies due to inconsistent definitions of metabolic health. MHO tends to be more common among women, younger individuals, those with lower BMI (<35 kg/m^2^), and people of European ancestry. However, MHO is often a transient state, with a significant proportion of individuals transitioning to MUO over time, underscoring the dynamic and progressive nature of obesity [26,27,28,29,30,31].

## 5. The Pathophysiology of Obesity

Obesity is a chronic, relapsing, multifactorial disease. It results from interactions among genetic susceptibility, neurohormonal and metabolic disturbances, behavioral factors, gut microbiota alterations, aberrant epigenetic modifications, and obesogenic environments characterized by hyperpalatable diets, easy access to high-calorie foods, overindulgence, and sedentary lifestyles. Accordingly, obesity extends beyond simple caloric excess or individual behavior, reflecting complex disruptions in the biological systems that regulate energy balance [32,33,34]. Indeed, energy homeostasis depends on hypothalamic and peripheral signals that coordinate intake and expenditure, and even small, sustained positive energy imbalances, potentially as little as 1–2% of daily energy intake, may contribute to substantial long-term weight gain [32,35].

At the tissue level, the sustained positive energy balance promotes triglyceride storage in adipocytes distributed across subcutaneous, visceral, pelvic, pericardial, and intramuscular depots. White adipose tissue (WAT) primarily stores energy, whereas the mitochondria-rich brown (BAT) and beige (BeAT) adipose tissues support thermogenesis [34,36]. Through uncoupling protein 1 (UCP1)-mediated uncoupled respiration, BAT and BeAT dissipate energy as heat; WAT browning can therefore increase expenditure and limit fat accumulation. Conversely, mitochondrial dysfunction and high-fat diets impair thermogenesis, promote insulin resistance, and aggravate metabolic complications [34].

Beyond its effects on thermogenesis, mitochondrial dysfunction also increases reactive oxygen species (ROS) production, activating inflammatory pathways that contribute to the chronic low-grade inflammation and insulin resistance associated with obesity [37,38]. In addition, mutations, deletions, or reduced copy number of mitochondrial DNA (mtDNA) can compromise energy production, increase oxidative stress, and lead to impaired glucose homeostasis, a key feature of obesity-related metabolic dysfunction [39,40].

Peripheral metabolic disturbances also influence neural circuits governing energy homeostasis. As a key integrative center, the hypothalamic arcuate nucleus (ARC) processes hormonal, metabolic, and neural cues to coordinate appetite and energy expenditure. Neurons co-expressing neuropeptide Y (NPY) and agouti-related peptide (AgRP) are orexigenic; they are stimulated by ghrelin and inhibited by leptin, insulin, amylin, and serotonin. AgRP promotes food intake by antagonizing the melanocortin-4 receptor (MC4R), thereby opposing α-melanocyte-stimulating hormone (α-MSH) signaling, reducing satiety, and stimulating overeating. Conversely, proopiomelanocortin (POMC) and cocaine- and amphetamine-regulated transcript (CART) neurons suppress appetite and increase energy expenditure. POMC-derived α-MSH activates MC4R to promote satiety, while CART also participates in reward-related neural circuits and behavioral responses to psychostimulants such as cocaine and amphetamine. In obesity, altered hormonal inputs and central resistance to leptin and insulin disrupt the balance between orexigenic NPY/AgRP and anorexigenic POMC/CART pathways [41].

Peripheral hormones relay nutrient availability and energy stores through the gut–brain axis. Ghrelin, secreted by the stomach during fasting periods, stimulates orexigenic neurons in the ARC, thereby promoting food intake, whereas leptin, produced by adipose tissue, engages leptin receptors (LEPR) within the ARC to inhibit hunger and enhance energy expenditure. Postprandial enteroendocrine hormones, including cholecystokinin (CCK) and GLP-1, signal to hypothalamic and brainstem circuits through vagal afferent and humoral pathways, promoting satiety and limiting meal size. In obesity, impaired leptin transport and signaling, hypothalamic inflammation and cellular stress, altered ghrelin signaling, and reduced responsiveness to satiety hormones, including CCK and GLP-1, sustain hunger and reduce energy expenditure [42,43,44]. Furthermore, excess adiposity, particularly visceral adiposity, together with persistent nutrient excess, impairs insulin signaling in skeletal muscle and liver, reducing glucose utilization and promoting compensatory hyperinsulinemia, hepatic lipogenesis, and continued weight gain [43,44].

## 6. Limitations of Traditional Nutritional Approaches in Obesity and the Emergence of Precision Nutrition

Traditional nutrition-based strategies for managing obesity typically center on reducing caloric intake and standardized meal plans. While these methods can promote initial weight loss, their long-term effectiveness is often limited due to interindividual metabolic differences, physiological adaptations, and difficulties sustaining adherence [4,45,46]. These limitations are particularly evident with low-energy diets (LEDs) and very-low-energy diets (VLEDs). While these regimens can induce rapid weight loss, they may also reduce fat-free mass, which is important for metabolic function and long-term weight control. VLEDs may achieve greater initial weight loss, but their long-term outcomes are generally no better than those of less restrictive approaches, and weight is frequently regained once dietary rigidity decreases. Highly restrictive regimens, including VLEDs and ketogenic diets, are further limited by inflexibility, potential nutritional insufficiencies, lifestyle barriers, and health risks such as gallstones and endocrine disturbances. In contrast, more flexible dietary patterns, such as the Mediterranean diet, tend to support better adherence and more sustainable long-term outcomes [45].

Beyond these practical challenges, the “calories in, calories out” framework provides an incomplete account of weight management. It assumes that energy intake and expenditure can be measured precisely and directly linked to changes in body weight. However, food labels may substantially misrepresent calorie content, sometimes by up to 60%, making precise energy balance difficult to achieve. Moreover, foods with the same energy content may have different metabolic effects: low-glycemic, fiber-rich foods promote satiety and slower glucose absorption, whereas high-glycemic foods can cause rapid insulin rises, favor fat storage, and lead to an earlier return of hunger. Thus, food quality influences weight management beyond total caloric intake. The framework also fails to fully capture adaptive physiological responses to energy restriction. Reduced energy intake can lower basal or resting metabolic rate through adaptive thermogenesis, thereby conserving energy during a perceived deficit. Genetic factors, insulin sensitivity, body composition, hormonal profiles, sleep, stress, and activity levels further influence metabolic responses to caloric restriction, limiting the predictive value of a simple caloric model and helping explain why identical dietary interventions may produce different outcomes [4].

Consequently, maintaining weight loss remains challenging even after successful short-term dietary interventions, with only around one in four individuals sustaining weight loss over time. Long-term maintenance is more likely among those who remain physically active, follow a balanced lower-fat diet, eat breakfast regularly, monitor their weight, and maintain consistent eating routines. Environmental management, self-regulation, structured therapeutic strategies, and continued behavioral support are also central to long-term success. Self-monitoring tools, including digital applications, can further strengthen adherence and help sustain these behavioral changes during the maintenance phase [46].

Taken together, these limitations emphasize why “one-size-fits-all” dietary approaches are insufficient in the management of obesity. Even when individuals follow the same standardized low-calorie, high-protein, or low-carbohydrate diet, they may show markedly different postprandial glucose responses to the same foods, as well as different changes in body composition, metabolic adaptations, and levels of adherence. This interindividual variability has driven the emergence of precision nutrition as a more individualized approach to obesity management [4,47].

## 7. Pillars of Individually Tailored Dietary Interventions: The Precision Nutrition Approach

Precision nutrition, also referred to as personalized nutrition, tailors dietary recommendations to individual biological, behavioral, and environmental characteristics, recognizing that responses to the same diet vary and that population-level guidelines may be insufficient for managing and preventing obesity and metabolic diseases. To create individualized interventions, precision nutrition integrates diverse factors such as genetic predisposition, physical activity, circadian rhythms, eating behaviors, metabolic status, and gut microbiome composition. Omics technologies, including genomics, epigenomics, transcriptomics, proteomics, metabolomics, lipidomics, foodomics, and microbiomics, provide a holistic assessment of biological systems and help identify interindividual differences in nutrient metabolism, food-derived metabolites, and metabotypes associated with metabolic risk. Personalized dietary guidance may enhance motivation for behavioral change and lead to superior outcomes in weight management compared with standard, non-personalized approaches [47,48,49,50].

### 7.1. Genetic Profiling for Dietary Recommendations

Obesity arises from a complex interaction between genetic predisposition and environmental exposures, with research indicating that 40–70% of individual variability in body weight is influenced by hereditary factors. From a genetic perspective, obesity can be classified as monogenic or polygenic. While the majority of obesity cases are polygenic, rare monogenic forms provide crucial insights into the fundamental biological pathways regulating appetite and energy balance [49,51].

#### 7.1.1. Monogenic Obesity and Specific Therapies

Monogenic obesity typically affects roughly 1–5% of individuals with severe early-onset obesity and is characterized by hyperphagia and rapid weight gain from early childhood. This condition usually results from rare, high-penetrance mutations in single genes that alter the leptin-melanocortin signaling pathway in the hypothalamus. The causal role, biological pathway, and molecular function of these genes have already been established through direct biochemical, physiological, and pedigree-based studies, independent of, and prior to, the GWAS that later identified common risk variants with much smaller individual effects. Key causal genes include leptin (*LEP*), *LEPR*, *POMC*, proprotein convertase subtilisin/kexin type 1 (*PCSK1*), and *MC4R*, each with a well-defined function within the leptin-melanocortin signaling pathway, as summarized in Table 2. In addition, obesity is a prominent clinical feature in several pleiotropic genetic syndromes, such as Prader–Willi syndrome and Bardet-Biedl syndrome [49,51].

Identifying monogenic obesity is clinically significant because it enables highly targeted, precision pharmacotherapy that addresses the underlying genetic defect, whereas conventional dietary interventions alone yield limited and often insufficient responses in these patients. A landmark example of this translational approach is setmelanotide (IMCIVREE^®^), an MC4R agonist approved by the FDA for chronic weight management in patients with specific genetic defects in the leptin-melanocortin pathway. Setmelanotide is indicated for individuals with obesity due to POMC, PCSK1, or LEPR deficiency, as well as for patients with Bardet-Biedl syndrome. By restoring signaling through the MC4R pathway, this targeted therapy effectively reduces hyperphagia and promotes significant weight loss, representing a paradigm shift toward precision medicine in obesity care [56]. Similarly, patients with congenital leptin deficiency due to biallelic *LEP* mutations respond markedly to recombinant human leptin (metreleptin) replacement therapy, which reduces hyperphagia and body weight and improves metabolic parameters, further illustrating the value of genotype-directed treatment in monogenic obesity [54,57].

#### 7.1.2. Polygenic Obesity

The more prevalent polygenic obesity arises from the combined small effects of numerous common gene variants, distinct from the rare, high-penetrance mutations previously discussed in the case of monogenic obesity. In the case of polygenic obesity, behaviors and living conditions, including eating patterns, exercise levels, and psychosocial factors, are key drivers in shaping the expression of genetic vulnerability [49,51,58,59]. GWAS have significantly expanded the understanding of polygenic obesity by identifying numerous loci across the genome that shape energy regulation, food intake behavior, fat accumulation, and variability in weight-loss outcomes. Obesity susceptibility is influenced by many genetic variants, particularly single nucleotide polymorphisms (SNPs) [60,61,62]. Notably, such associations are often population-specific: the rs9939609 variant of the fat mass and obesity-associated gene (*FTO*), robustly linked to obesity in European cohorts, showed no association in a Chinese Han population, underscoring that SNP-based findings should not be generalized across ethnic groups without validation [63].

Table 3 summarizes some of the most relevant and well-replicated SNPs statistically associated with susceptibility to obesity in GWAS. Unlike the causal monogenic genes described above, whose functional roles were established through direct biochemical and physiological study, the mechanistic annotations below reflect the biological plausibility proposed for each locus and should not be read as proof of a validated causal or functional role. The *FTO* gene is known for its influence on appetite and body weight, and variants in *MC4R* are similarly associated with food intake, distinct from the rare loss-of-function *MC4R* mutations that cause monogenic obesity (Table 2). Variants in the *LEP* gene are associated with differences in appetite control and metabolic homeostasis. Likewise, the peroxisome proliferator-activated receptor gamma (*PPARG*) gene, important for adipocyte differentiation and fat and glucose metabolism, and the brain-derived neurotrophic factor (*BDNF*) gene, which regulates appetitive behavior and energy balance, harbor common variants associated with obesity risk. Similarly, common variants within the apolipoprotein A-II (*APOA2*), fatty acid-binding protein 2 (*FABP2*), and adrenoceptor beta 2 (*ADRB2*) genes, involved, respectively, in gene–diet interactions with saturated fat intake, intestinal dietary fat absorption, and adrenergic-mediated energy expenditure, further help explain the wide range of obesity-related responses among individuals [51,64,65,66,67,68,69,70].

The integration of genome-wide SNP data into personalized dietary recommendations represents an emerging strategy in the management of obesity [82]. Some large-scale trials have reported encouraging results in this direction. In the multinational Food4Me trial, participants informed of their *FTO* obesity-risk-allele status as part of a genetically personalized intervention showed significantly greater reductions in body weight and waist circumference than non-risk-allele carriers within the same intervention arm [83]. Likewise, in the NOW trial, incorporating nutrigenomics-based guidance into a structured lifestyle program yielded significantly greater short- and medium-term reductions in body fat percentage than the standard program alone [84].

Nevertheless, other studies specifically testing whether matching diet composition to genotype, rather than simply disclosing genetic risk or adding nutrigenomic guidance to standard counseling, outperforms standard advice have shown more limited clinical evidence. For instance, the Personalized Nutrition Study (POINTS) study classified 145 adults with overweight or obesity as fat- or carbohydrate-responders based on ten SNPs and randomized them to a high-fat or high-carbohydrate diet, resulting in genotype-concordant and genotype-discordant subgroups. There was no significant difference in weight loss between these subgroups (−5.3 kg vs. −4.8 kg) after 12 weeks [85]. Likewise, the MyGeneMyDiet study compared genotype-informed lifestyle recommendations against standard dietary advice in Filipino adults with overweight and obesity over 12 months, and found no evidence of meaningful between-group differences in weight, BMI, waist circumference, body fat percentage, or metabolic markers at any timepoint, although the small final sample size (27 completers out of 52 who initiated the intervention) may limit the strength of these results [86].

These findings are consistent with a broader pattern in the literature: while the theoretical framework for genotype-guided dietary intervention is well-established, the translation of SNPs data into clinically meaningful and superior outcomes over standard advice remains largely unproven for approaches specifically matching diet type to genotype. It is also important not to conflate statistical association with functional validation: a GWAS hit indicates that a locus is statistically linked to obesity risk in a given population, not that the underlying variant has been experimentally shown to cause the phenotype through the proposed mechanism. Several factors contribute to the limited clinical translation of these findings. First, individual SNPs and even multi-SNP genetic risk scores typically explain only a modest fraction of the variance in BMI and adiposity, an effect size consistent with the minimal, largely non-actionable contribution of any single common variant, given that obesity is a highly polygenic and environmentally modulated trait. Second, current SNP panels do not yet capture the full complexity of nutrient metabolism, which is further shaped by aberrant epigenetic modifications, the gut microbiome, metabolic phenotype, and behavioral factors that static genomic data alone cannot reflect. Therefore, progress in the field will require the integration of multi-omics approaches, larger GWAS datasets, and rigorously designed randomized controlled trials before genotype-guided nutrition can be implemented as a standard clinical tool in obesity [49,86,87].

One such underexplored multi-omics direction involves tracing genetic variation back to the enzymatic steps of carbohydrate, protein, and lipid metabolism themselves, rather than stopping at associations between SNPs and downstream phenotypes such as body weight. SNPs affecting the activity of specific metabolic enzymes may offer greater explanatory value for individual dietary responses than SNPs associated only with such distal phenotypic effects. The mitochondrial dysfunction, discussed in Section 5, can be understood in this context as a downstream signal of upstream disruptions in these biochemical pathways rather than a primary cause in itself. For instance, excess branched-chain amino acid availability, reflecting altered protein catabolism, has been shown to suppress mitochondrial function and biogenic signaling in a skeletal muscle model of insulin resistance, illustrating how a specific macronutrient-metabolizing pathway can drive mitochondrial impairment [88]. Greater attention to causal genes involved in macronutrient metabolism may therefore complement current SNP-based approaches to precision nutrition.

### 7.2. Metabolomic-Guided Dietary Adjustments

Metabolomics, one of the major omics disciplines, analyzes low-molecular-weight metabolites that represent intermediate or final products of biochemical pathways at cellular and systemic levels. By integrating both intrinsic host processes and influences from digestion, nutrient metabolism, and gut microbiota activity, the metabolomic profile provides a broad overview of an individual’s overall physiological state. In obesity, it can reveal molecular alterations associated with excess adiposity and identify biomarkers of metabolic dysregulation, including impaired glucose homeostasis and insulin resistance [89,90].

A compelling illustration of metabolomics applied in interventions to combat obesity is provided by the POUNDS Lost trial, a 2-year randomized controlled trial in 811 adults with overweight or obesity assigned to four diets varying in protein and fat content. Metabolomic analyses within this trial revealed that reductions in circulating branched-chain amino acids (valine, leucine, isoleucine) and aromatic amino acids during the intervention were significantly associated with improvements in insulin resistance and reductions in triglycerides, independent of the amount of weight lost. These findings suggest that metabolomic profiling can identify metabolic responders to dietary intervention beyond what anthropometric measures alone can capture, supporting the use of metabolomics to monitor and guide precision dietary strategies in obesity management [91]. Accordingly, the following subsections examine two complementary dimensions of this metabolic heterogeneity: personalized glycemic responses and lipidomic alterations related to fatty acid metabolism.

#### 7.2.1. Personalized Glycemic Response

Personalized glycemic response describes the substantial interindividual variability in postprandial glycemic responses (PPGRs) after identical meals, challenging the assumption that foods exert uniform glycemic effects based on carbohydrate content or glycemic index alone. Frequent postprandial glucose (PPG) elevations are associated with altered glucose homeostasis, hyperinsulinemia, and obesity-related metabolic dysfunction [92]. Elevated PPG stimulates insulin secretion and may promote weight gain, whereas well-regulated PPG may preserve insulin sensitivity, improve satiety, and support weight loss. Given the considerable variability in these responses among individuals, standardized nutritional guidelines may not adequately reflect personal metabolic needs [93]. This concept is most directly actionable for individuals with, or at high risk of, dysglycemia and type 2 diabetes mellitus. Therefore, glycemia-guided personalization should be regarded as one tool within a broader personalization strategy rather than a universal requirement of obesity care [94].

A first source of variability in PPGRs lies in meal composition and sequencing rather than carbohydrate content alone. In a controlled trial of standardized carbohydrate meals (rice, bread, potatoes, pasta, beans, grapes, and mixed berries) with equivalent glycemic loads, postprandial responses varied substantially between individuals, and consuming small amounts of fiber, protein, or fat before carbohydrate intake modestly blunted glycemic peaks in many participants, underscoring the role of nutrient composition and food characteristics in modulating glycemic response beyond carbohydrate quantity alone. A second, individual-level source is the person’s own metabolic phenotype: within the same trial, participants with greater insulin resistance or pancreatic β-cell dysfunction showed higher glucose responses to starch-rich foods such as potatoes and pasta, and the benefit of meal sequencing was blunted in these individuals, indicating that food-based strategies interact with, rather than override, the underlying metabolic phenotype [92].

A third factor contributing to variability in glycemic responses is the gut microbiota. Gut microbial composition and functional activity may influence carbohydrate metabolism and PPGRs via fermentation metabolites, inflammatory pathways, and insulin signaling. Thus, incorporating microbiota features into predictive models may improve glycemic response estimation beyond conventional dietary approaches [90].

Clinical evidence increasingly supports the value of glycemia-guided personalization: continuous glucose monitoring-guided nutrition counseling produced significantly greater reductions in body weight, BMI, body fat mass and waist-to-hip ratio than conventional advice over 12 weeks in adults with obesity [93]. Likewise, Zeevi et al. developed and validated a ML algorithm integrating diet, physical activity, anthropometrics, blood parameters, and gut microbiota composition to predict PPGRs, and showed in a small randomized trial that algorithm-guided diets reduced glucose spikes alongside favorable microbiota shifts [95]. Applying the Zeevi et al. algorithm [95], the Personal Diet Study found comparable six-month weight loss (approximately 3–4%) between a standardized low-fat diet and the algorithm-guided intervention in adults with obesity and impaired glucose metabolism, although individual responses varied considerably, suggesting some individuals may derive greater benefit from personalized strategies than others [96].

More recently, the ZOE METHOD trial compared, among 347 US adults, an 18-week app-based personalized dietary program, based on food characteristics, individual postprandial glucose and triglyceride responses, gut microbiome composition, and health history, against standard US Department of Agriculture-based dietary advice, and found significantly greater reductions in triglycerides, along with secondary improvements in body weight, waist circumference, and microbiome diversity, particularly among highly adherent participants. However, as this trial was conducted in a generally healthy population rather than an obesity-specific cohort, its findings should not be over-generalized to individuals with obesity [97].

Taken together, glycemic responses to food reflect an interplay of meal composition, individual metabolic phenotype, and gut microbial characteristics, and predictive tools accounting for this complexity may improve the personalization of dietary strategies for obesity management. Although current evidence is encouraging, further long-term trials are needed to clarify which individuals benefit most from personalized glycemic interventions and whether these approaches translate into clinically meaningful, sustained weight loss.

#### 7.2.2. Lipidomics and Fatty Acid Metabolism

Lipidomics may provide a more detailed assessment of obesity-related cardiometabolic dysfunction than conventional lipid panels by characterizing individual lipid species and metabolic pathways [98,99]. Obesity-associated lipidomic profiles commonly show increased saturated lipid metabolites, reduced unsaturated fatty acids, particularly polyunsaturated fatty acids (PUFAs), and altered phospholipid metabolism, changes associated with insulin resistance, visceral adiposity, hypertriglyceridemia, and persistent low-grade inflammation [100]. Reduced lysophosphatidylcholine and phosphatidylcholine species may reflect disturbances in choline-dependent one-carbon metabolism, epigenetic regulation, and energy homeostasis, while enzymes such as PHOSPHO1 connect phospholipid turnover with insulin sensitivity and adipose-tissue thermogenesis [101]. As some abnormalities may precede overt obesity or conventional dyslipidemia, lipidomic biomarkers may support early risk assessment and personalized nutrition [98].

Furthermore, fatty acid composition and metabolism are central to obesity-related metabolic dysfunction. Saturated fatty acids (SFAs), especially palmitic acid, promote inflammation, impaired insulin signaling, hepatic steatosis, and lipotoxicity. Likewise, combined high intakes of SFAs and simple carbohydrates can increase ceramide synthesis, which is linked to insulin resistance, mitochondrial dysfunction, and metabolic inflammation. The broader relevance of this pathway is reflected in its involvement in sphingolipid lysosomal storage disorders [102]. In contrast, monounsaturated fatty acids (MUFAs) and PUFAs are generally associated with improved membrane function, enhanced insulin sensitivity, and more efficient fatty acid oxidation, via carnitine shuttle-dependent mitochondrial and peroxisomal pathways; dietary fatty acid composition itself directly regulates mitochondrial structure and function, indicating that the metabolic impact of dietary fat depends on fatty acid composition and its subsequent oxidative processing, and not simply on total fat intake [103,104,105].

Numerous dietary intervention studies have demonstrated that modifying the type of fat consumed favorably influences lipidomic profiles and metabolic outcomes in obesity. Diets rich in oleic acid, the main MUFA in olive oil, reduce ceramide concentrations and enhance insulin sensitivity compared with diets high in palmitic acid and other SFAs. Likewise, overfeeding studies show that saturated fat-rich diets promote ceramide accumulation and liver fat, whereas PUFA-enriched diets preserve a more favorable lipid profile despite similar weight gain. Foods naturally rich in unsaturated fatty acids, including extra virgin olive oil, fatty fish, and walnuts, may similarly lower circulating ceramides and other lipid intermediates involved in inflammation and insulin resistance [103,106,107,108,109].

Omega-3 PUFAs, particularly eicosapentaenoic acid (EPA) and docosahexaenoic acid (DHA), may stimulate fat oxidation, support mitochondrial function, improve insulin sensitivity, while reducing hepatic lipid accumulation and pro-inflammatory mediator production. Conversely, a high omega-6-to-omega-3 ratio may promote metabolic dysregulation, making this dietary balance relevant to metabolic health in obesity [110]. Finally, genetic factors and variation in fatty acid-metabolizing enzymes may influence PUFAs metabolism, insulin sensitivity, inflammatory status, and lipid profiles. Lipidomic profiling may therefore identify metabolic phenotypes and guide individualized interventions, including targeted omega-3 supplementation and personalized modifications in fat intake [111].

### 7.3. Microbiome-Targeted Interventions

*Bacillota* (formerly *Firmicutes*) and *Bacteroidota* (formerly *Bacteroidetes*) are the dominant phyla in the gut microbiota, while *Pseudomonadota* (formerly *Proteobacteria*), *Verrucomicrobiota* (formerly *Verrucomicrobia*), and *Actinomycetota* (formerly *Actinobacteria*) are found in smaller amounts, together contributing to nutrient assimilation, immune regulation, and host metabolic functions. Obesity is frequently associated with reduced microbial diversity and an increased *Bacillota* to *Bacteroidota* ratio, although this finding remains inconsistent [112,113,114]. Independently of this ratio, obesity-associated dysbiosis has been linked to reduced abundance of taxa such as *Akkermansia muciniphila* and *Faecalibacterium prausnitzii*, along with functional shifts in short-chain fatty acid (SCFA) production, bile acid signaling, and intestinal barrier integrity, which may contribute to impaired glucose regulation and persistent low-grade inflammation [115,116]. Obesity-associated dysbiosis has also been linked to decreased intestinal expression of fasting-induced adipose factor (Fiaf) and impaired signaling of anorexigenic gut hormones like GLP-1 and peptide YY (PYY), which may promote energy intake and lipid storage [117]. These findings have prompted interest in probiotics, prebiotics, and fecal microbiota transplantation to modulate the gut microbiota in obesity management [118].

#### 7.3.1. Probiotics and Prebiotics

Probiotics are viable microorganisms that confer health benefits when ingested in adequate amounts. Strains of *Lactobacillus* and *Bifidobacterium* may influence body weight through production of SCFA from fiber fermentation, upregulation of satiety hormones (GLP-1, PYY, CCK, leptin), suppression of the “hunger hormone” ghrelin, gut–brain signaling, and restoration of gut barrier integrity. Evidence is strongest for SCFAs-mediated stimulation of GLP-1 and PYY, while overall efficacy depends on strain, dosage, duration of supplementation, and host characteristics [114,118,119,120].

Clinical evidence supports modest but meaningful benefits of probiotic supplementation in obesity management. *Lactobacillus gasseri* SBT2055, one of the most studied strains, reduced body weight, BMI, waist circumference, and visceral fat when consumed as fermented milk or yoghurt (100–200 g/day) for 12 weeks compared to placebo. Similar outcomes have been reported for other *Lactobacillus* and *Bifidobacterium* strains over 6–12 week interventions, whereas shorter trials of 3–4 weeks were generally ineffective [114,117,121,122,123].

Multi-strain probiotic formulations may yield more consistent results. A six-month RCT using a five-strain combination (*L. acidophilus* CUL60, CUL21; *L. plantarum* CUL66; *B. bifidum* CUL20; *B. animalis* subsp. *lactis* CUL34; 5 × 10^10^ CFU/capsule) achieved a mean weight reduction of ~1.3 kg with improvements in BMI and body composition [124], consistent with meta-analytic estimates of modest reductions in body weight (~0.5–0.6 kg), BMI (~0.3 kg/m^2^), and fat mass, more pronounced beyond eight weeks and when combined with caloric restriction or exercise [120]. Notably, while strains such as *L. gasseri* SBT2055 promote weight loss, others such as certain *L. acidophilus* strains may paradoxically increase weight by altering carbohydrate metabolism, underscoring the need for individualized strain selection [114].

Prebiotics, including inulin, fructo-oligosaccharides (FOS), and galacto-oligosaccharides (GOS), are indigestible dietary compounds that selectively stimulate beneficial gut bacteria. In obesity management, their fermentation into SCFAs may enhance satiety by stimulating PYY and GLP-1 secretion and potentially reducing circulating ghrelin. SCFAs also support intestinal barrier integrity and may attenuate metabolic endotoxemia and the chronic low-grade inflammation characteristic of metabolic syndrome [113,119,125]. However, prebiotic efficacy varies according to baseline gut microbiota composition. Inulin-type fructans, for instance, show heterogeneous metabolic effects depending on the pre-existing microbial ecosystem, making standardized protocols difficult to establish. Metagenomic profiling may therefore enable the development of targeted fiber formulations tailored to individual gut ecology, supporting precision nutrition approaches [126,127,128,129].

#### 7.3.2. Fecal Microbiota Transplantation

Fecal microbiota transplantation (FMT) involves transferring gut microorganisms from a healthy donor to a recipient, typically via duodenal endoscopy or colonoscopy, to restore microbial diversity and metabolic homeostasis [125]. Clinical studies indicate that FMT from lean donors may improve gut barrier function, insulin sensitivity, metabolic parameters, and bile acid metabolism in individuals with obesity, partly through increased abundance of *Faecalibacterium* species [130,131]. Although its effects on BMI are generally limited, a four-year follow-up study in adolescents showed sustained reductions in waist circumference, body fat, and systemic inflammation, alongside increased HDL-cholesterol and persistent microbiome changes [132]. However, efficacy varies with donor microbial profile, recipient characteristics, and treatment protocols. Overall, FMT appears more promising as a complementary strategy for improving metabolic health and inflammation than as a primary weight loss intervention [130].

### 7.4. Chrono-Nutrition: Timing of Food Intake as a Personalization Target

Beyond the quantity and quality of the diet, growing evidence indicates that the timing of food intake independently influences body weight and metabolic health, giving rise to the field of chrono-nutrition. Time-restricted eating (TRE), which confines daily food intake to a fixed window without necessarily prescribing caloric restriction, has been the most extensively studied approach in this context. In a landmark trial, a 10 h TRE window reduced body weight, blood pressure, and atherogenic lipids in adults with metabolic syndrome. A systematic review and meta-analysis focusing specifically on adults with obesity similarly found that TRE produced reductions in body weight and fat mass. More broadly, a recent network meta-analysis of randomized clinical trials comparing multiple intermittent fasting strategies confirmed clinically meaningful reductions in body weight and several cardiometabolic risk factors, although effects were generally comparable to those achieved with continuous caloric restriction, suggesting that part of the benefit may stem from spontaneous reductions in energy intake rather than meal timing per se [133,134,135].

A key limitation of current chrono-nutrition research is that most intervention protocols apply a fixed eating window uniformly across participants, without accounting for individual circadian biology. Chronotype, the behavioral manifestation of an individual’s intrinsic circadian phase, is influenced by genetic variation in core clock genes and determines each person’s natural propensity toward morningness or eveningness. This, in turn, influences appetite regulation, glucose tolerance, and the metabolic response to meal timing throughout the day. Individuals with an evening chronotype, for instance, tend to exhibit poorer glycemic control and greater difficulty adhering to early time-restricted eating protocols than those with a morning chronotype. These findings suggest that aligning the timing of food intake with an individual’s chronotype, rather than applying a standardized eating window, may represent an underexplored but promising dimension of precision nutrition, warranting integration alongside genetic, metabolomic, and microbiome profiling as part of a comprehensive personalization strategy [136,137].

### 7.5. Behavioral and Lifestyle Integration

Behavioral and lifestyle factors constitute essential components of precision nutrition, as the long-term success of obesity management depends not only on an individual’s biological characteristics but also on their ability to adopt and sustain meaningful changes in everyday habits. Personalized dietary interventions are therefore more likely to be effective when they are aligned with personal food preferences, cultural practices, psychosocial circumstances, and daily routines, including physical activity patterns, sleep habits, and stress management [47]. Psychological characteristics such as motivation, self-efficacy, and self-regulation further influence the capacity to maintain these changes over time [138].

A wide range of behavioral techniques has been shown to support adherence to personalized interventions. Common behavioral strategies include monitoring dietary intake, physical activity, and body weight to improve self-awareness and support ongoing commitment to healthier behaviors, together with setting achievable goals that promote long-term change. Additional strategies involve problem-solving techniques designed to help individuals overcome obstacles that may interfere, stress management to reduce emotionally driven eating, and stimulus control, which focuses on modifying environmental factors that encourage overeating or sedentary behavior. Support from family, friends, and healthcare professionals has also been associated with improved weight-loss maintenance [139].

Recent evidence has further emphasized the importance of addressing the psychological mechanisms underlying eating behavior. Food cravings are influenced by neurobiological reward pathways and conditioned responses to highly palatable foods, which may trigger eating in the absence of physiological hunger. Educating individuals about these processes can reduce self-blame and facilitate the development of more effective coping strategies. Approaches grounded in acceptance and commitment may provide additional benefit by strengthening the ability to tolerate discomfort, manage cravings, and maintain behaviors that are consistent with personal values and long-term health goals [138].

Lifestyle-related factors are equally important for achieving and preserving weight loss. Adequate and consistent sleep has been associated with improved appetite regulation and a lower likelihood of weight regain, whereas regular physical activity contributes to weight maintenance, preservation of lean body mass, and improvements in both metabolic and psychological health. Comprehensive interventions generally recommend at least 150 min per week of moderate-intensity aerobic exercise during the initial phase of weight loss, with higher levels of physical activity, approximately 200 to 300 min per week, often suggested to support long-term weight maintenance. Reducing sedentary time and incorporating movement into daily routines may further enhance the sustainability of personalized obesity treatment strategies [140,141].

### 7.6. Enhancing GLP-1 Efficacy with Personalized Nutrition

#### 7.6.1. GLP-1 and Dual GIP/GLP-1 Receptor Agonists in Obesity Management

Currently available pharmacological therapies for the management of obesity include several agents that act through distinct mechanisms. Orlistat is a gastrointestinal lipase inhibitor that reduces dietary fat absorption, but its use is often limited by gastrointestinal adverse effects, including oily fecal spotting and urgency in more than 25% of patients, along with decreased absorption of fat-soluble vitamins. Phentermine-topiramate and naltrexone-bupropion act on the central nervous system to suppress appetite but are constrained by neuropsychiatric and cardiovascular effects, including insomnia, paresthesia, and teratogenicity with topiramate, and contraindication in seizure disorders and uncontrolled hypertension with naltrexone-bupropion [142].

More recently, obesity therapies targeting gut-derived hormones have emerged, producing substantially greater weight loss in head-to-head comparisons against placebo (semaglutide, 11.4%; tirzepatide, 12.4%) than orlistat (3.1%), naltrexone-bupropion (4.1%), or phentermine-topiramate (8.0%), while also demonstrating a broader range of systemic benefits, including glucometabolic, cardiovascular, hepatic, and renal effects, in dedicated outcome trials. This combination of superior efficacy and pleiotropic health benefits has positioned gut hormone-based drugs, modeled on hormones known as incretins, as the current cornerstone of obesity pharmacotherapy [142,143].

Glucose-dependent insulinotropic polypeptide (GIP) and GLP-1 are the two primary incretin hormones in humans. GIP is secreted by K-cells, predominantly located in the duodenum and jejunum, whereas GLP-1 is secreted by L-cells, which are distributed mainly in the ileum and colon. L-cells also co-secrete PYY hormone, which reduces appetite and regulates glucose metabolism [144,145].

Together, GIP and GLP-1 mediate the incretin effect, a physiological phenomenon characterized by enhanced insulin secretion following oral glucose administration compared with an identical glucose load delivered intravenously, reflecting their central role in postprandial glycemic control. GIP and GLP-1 receptors are expressed in pancreatic, neural, gastrointestinal, adipose, and other metabolically relevant tissues, enabling effects beyond insulin secretion. GLP-1 inhibits glucagon secretion during hyperglycemia and euglycemia, but not during hypoglycemia, delays gastric emptying, and promotes satiety via central nervous system circuits. GIP, by contrast, has little effect on glucagon during hyperglycemia but promotes glucagon secretion under fasting, euglycemic, and hypoglycemic conditions. In adipose tissue, GIP facilitates postprandial lipid uptake and storage, partly by enhancing insulin-dependent lipoprotein lipase activity. Its role in appetite control has historically been considered modest relative to GLP-1, though preclinical evidence suggests central GIP receptor signaling may contribute to reductions in food intake [145,146].

These physiological actions of endogenous incretin hormones have been pharmacologically leveraged in the development of GLP-1 receptor agonists (RAs) (e.g., semaglutide, liraglutide), as well as newer dual GIP/GLP-1 RAs, including tirzepatide [147,148,149]. Building on this progression, triple-hormone agonists represent the next stage in incretin-based drug development. For instance, retatrutide, an investigational first-in-class triple GIP/GLP-1/glucagon receptor agonist, has recently demonstrated topline weight-loss results in the Phase 3 TRIUMPH-1 trial, with 45.3% of participants on the 12 mg dose achieving ≥30% weight loss at 80 weeks, a magnitude previously associated only with bariatric surgery. This positions retatrutide as a potentially transformative pharmacological option for obesity management, pending full peer-reviewed data and regulatory approval [150,151].

GLP-1 RAs and dual GIP/GLP-1 RAs are generally prescribed alongside lifestyle modification in individuals with a BMI ≥ 30 kg/m^2^ or a BMI ≥ 27 kg/m^2^ accompanied by at least one obesity-related comorbidity [152]. Beyond weight loss, emerging evidence suggests that these therapies provide beneficial effects across several obesity-associated comorbidities, including cardiovascular disease, chronic kidney disease, metabolic dysfunction-associated steatotic liver disease, and certain types of cancer [147,153,154,155,156].

At therapeutic doses, GLP-1 RAs amplify and prolong the GLP-1-mediated effects described above. Their principal anti-obesity effects include reduced appetite and hedonic eating, delayed gastric emptying, enhanced satiety, and lower food intake. They may also improve leptin sensitivity and energy expenditure. In addition, they improve glucose homeostasis through glucose-dependent insulin secretion and reduced glucagon release and may benefit lipid metabolism, adipose-tissue function, and systemic inflammation [157]. Interestingly, beyond their established metabolic effects, GLP-1 RAs show promise in addictive disorders, including alcohol use disorder, by modulating mesolimbic dopamine signaling and reward-driven craving [158,159].

In dual GIP/GLP-1 RAs, GIP receptor activation complements GLP-1 receptor signaling and may further improve glycemic regulation, lipid metabolism, adipose-tissue function, and metabolic flexibility. It may also facilitate switching between carbohydrate and fat utilization, potentially increasing energy expenditure. By targeting multiple pathways implicated in obesity and metabolic dysfunction, dual agonists may enhance appetite regulation, glucose metabolism, and body-weight control, helping explain their superior therapeutic efficacy relative to GLP-1 RAs alone [157,160].

Despite their therapeutic benefits, GLP-1 RAs and dual GIP/GLP-1 RAs are associated with several clinical challenges, including gastrointestinal adverse effects, interindividual variability in treatment response, loss of lean body mass, and the potential development of nutritional deficiencies. These limitations emphasize the importance of integrating personalized nutritional interventions to enhance treatment tolerability, support metabolic health, and improve long-term therapeutic success [147].

#### 7.6.2. Management of Gastrointestinal Adverse Effects

Gastrointestinal adverse effects represent the most commonly reported complications of GLP-1 RAs and dual GIP/GLP-1 RAs. The most frequent symptoms include nausea, vomiting, diarrhea, constipation, abdominal discomfort, bloating, and dyspepsia. These adverse events are typically mild to moderate, occur more frequently during dose escalation, and tend to diminish with continued treatment. Their development is largely attributed to delayed gastric emptying, altered gastrointestinal motility, and central mechanisms involved in appetite and nausea regulation. Higher therapeutic doses have also been associated with a greater likelihood of gastrointestinal intolerance [161].

Nausea is generally the most common gastrointestinal adverse effect during the early stages of GLP-1-based therapy, particularly during dose increase, and may be exacerbated by large or high-fat meals. Nutritional strategies that may relieve nausea include consuming smaller, more frequent meals, eating slowly, avoiding overeating, limiting foods with strong odors or high fat content (e.g., fried foods) and, when needed, consuming bland foods such as crackers or dry toast [6,161]. Ginger may provide additional symptomatic relief because of its antiemetic properties [162].

Vomiting is less common than nausea but, if persistent, may lead to dehydration, electrolyte imbalance, and reduced dietary intake. Patients experiencing vomiting are generally encouraged to prioritize adequate fluid intake and consume small amounts of food at shorter intervals to minimize gastric discomfort. Avoiding very large meals and foods that are difficult to digest may further improve symptom tolerability [6].

For diarrhea, nutritional management should primarily focus on maintaining adequate hydration and temporarily limiting foods that may worsen gastrointestinal symptoms, including alcohol, excessive caffeine, sugar alcohols, highly processed foods, and certain high-fiber products in individuals with lower gastrointestinal tolerance. In some patients, choosing more easily digestible foods and progressively reintroducing dietary fiber may support better gastrointestinal adaptation and symptom improvement over time [152,161].

Constipation may occur because of delayed gastric emptying, reduced gastrointestinal motility, lower food intake, and insufficient hydration. Personalized dietary approaches typically emphasize adequate fluid intake, gradual optimization of dietary fiber according to individual tolerance, and regular physical activity. Incorporating fiber-rich foods progressively, rather than abruptly increasing fiber consumption, may help minimize bloating and improve bowel regularity during therapy [6,152,161,162].

#### 7.6.3. Nutritional Considerations During GLP-1-Based Therapy

Lean body mass preservation and maintenance of adequate nutritional intake have emerged as important considerations during GLP-1 RAs and dual GIP/GLP-1 RAs therapy. Although these therapies are highly effective for weight reduction, their potent appetite-suppressing effects can substantially reduce spontaneous food intake and compromise macronutrient and micronutrient consumption. Nutritional management should therefore extend beyond calorie restriction alone and emphasize individualized energy intake, overall diet quality, balanced macronutrient distribution, adequate fiber and fluid intake, and prevention of nutritional inadequacies. Energy intake recommendations should be individualized according to age, sex, body weight, physical activity level, and treatment response, while excessively restrictive dietary approaches may increase the risk of dehydration, electrolyte imbalance, gallstone formation, and inadequate nutrient intake [152,163,164].

Weight loss achieved with GLP-1 RAs and dual GIP/GLP-1 RAs may involve reductions in lean body mass, although current evidence suggests that these changes are highly variable and do not necessarily reflect clinically meaningful deterioration in skeletal muscle function. Some studies indicate that improvements in muscle quality and insulin sensitivity may occur despite decreases in lean mass, suggesting that part of this change reflects an adaptive response to weight loss rather than clinically significant muscle deterioration [165]. Nevertheless, preserving lean body mass remains an important consideration, particularly in older adults and individuals at risk of sarcopenia [166]. Notably, bimagrumab, a recombinant human monoclonal antibody targeting activin type II receptors (ActRIIA and ActRIIB), is a promising therapeutic approach for reducing fat mass while preserving lean mass, both as monotherapy and in combination with GLP-1 RAs. Preclinical and phase 2 clinical evidence indicates that ActRII blockade may enhance fat loss and attenuate the reduction in lean mass associated with GLP-1 RA therapy [167,168].

Nevertheless, adequate, individualized protein intake and regular physical activity are key components of nutritional care during incretin-based therapy. While general recommendations suggest a protein intake of approximately 0.8 g/kg/day for adults, higher intakes ranging from 1.0 to 2.0 g/kg/day may be more beneficial during GLP-1-based treatment, especially in older adults and those with sarcopenic obesity. Distributing high-quality protein evenly across meals, with approximately 25–30 g per meal, may further support muscle protein synthesis. Adequate sources of protein include fish, poultry, yogurt, and legumes. In cases of severely reduced appetite, the use of high-protein oral nutritional supplements providing a minimum of 20 g of protein per serving may help meet protein requirements [147,162,169]. Resistance training, combined with regular aerobic physical activity, should complement these dietary measures to help preserve lean mass and improve muscle strength during weight-loss interventions. Current recommendations include at least 150 min of moderate-to-vigorous aerobic exercise weekly alongside resistance training comprising 2–3 weekly sessions of approximately 30 min each, using free weights or resistance bands [165,166].

Balanced carbohydrate and fat intake also remains important. Severe carbohydrate restriction does not appear necessary for long-term weight management and may unintentionally reduce the intake of fiber-rich foods, including fruits, vegetables, legumes, and whole grains, which provide important micronutrients and cardiometabolic benefits. Similarly, healthy dietary fats are important for the absorption of fat-soluble vitamins and maintenance of overall nutritional status during rapid weight loss. Emphasis should therefore be placed on unsaturated fat sources such as nuts, seeds, olive oil, avocado, and fatty fish, while limiting fried foods and foods rich in saturated fats that may exacerbate gastrointestinal symptoms [152].

Adequate dietary fiber intake and hydration further support gastrointestinal health, cardiometabolic regulation, and prevention of constipation. Fiber should be increased gradually and according to tolerance, with supplementation considered if dietary intake remains insufficient. Because reduced appetite and altered thirst perception may contribute to inadequate hydration, regular fluid intake, primarily water and low-calorie beverages, should be encouraged, while alcohol and excessive caffeine are limited [152,166].

Micronutrient status also requires attention, as individuals with obesity may have suboptimal intake before treatment and appetite suppression can exacerbate deficiencies. Patients receiving incretin therapies may fail to meet recommended intakes of vitamins A, D, E, K, and B-group vitamins, as well as calcium, iron, magnesium, and potassium. Observational studies report that a substantial proportion develop at least one deficiency within the first treatment year, with vitamin D status frequently affected [163,164,170,171].

**Figure 2:**
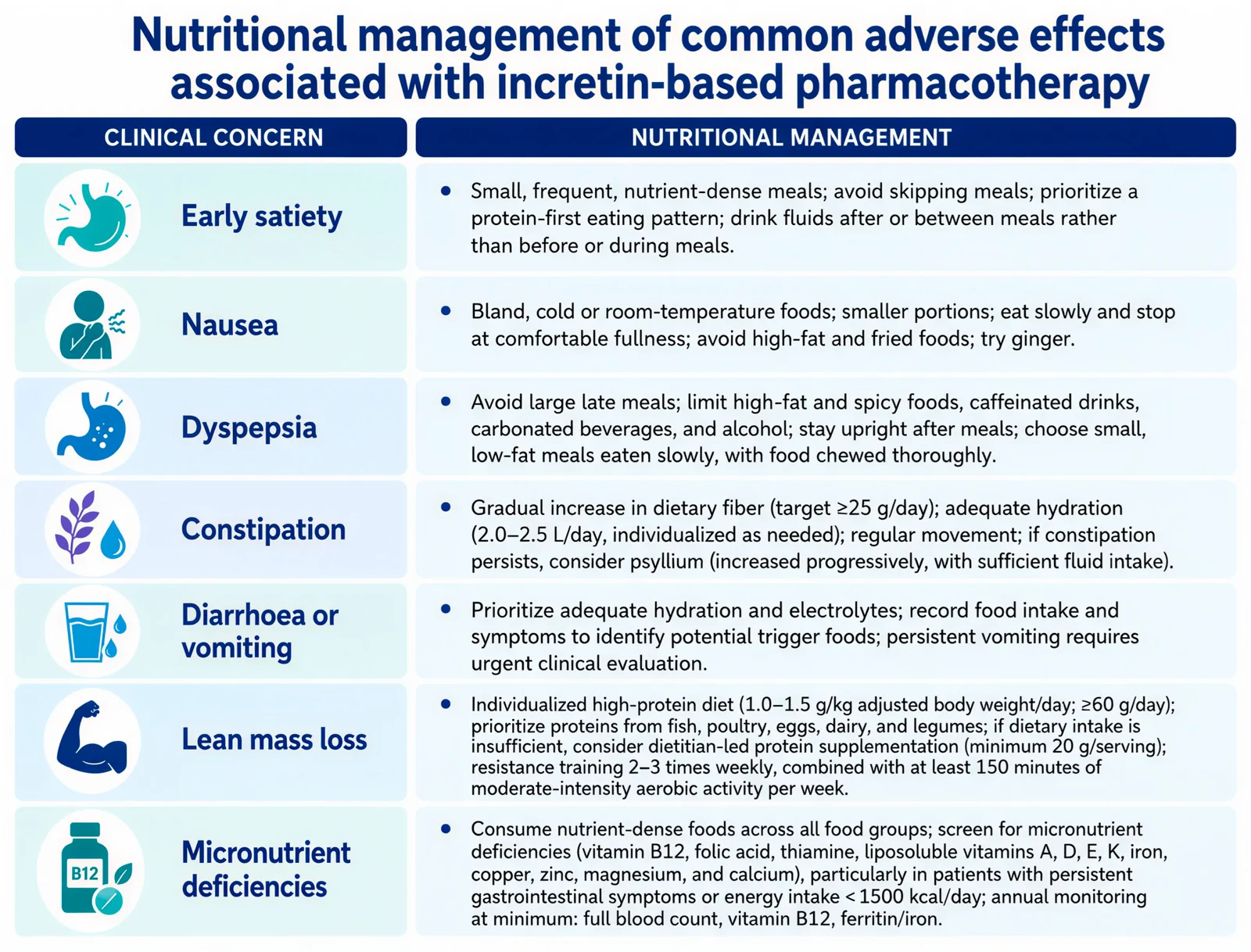
Nutritional management of common adverse effects associated with incretin-based pharmacotherapy (adapted from [6,162,166,172,173,174]).

Personalized dietary strategies should prioritize nutrient-dense, minimally processed foods and balanced meals despite lower energy intake. For reduced appetite, early satiety, or gastrointestinal discomfort, smaller, more frequent meals and soft or liquid nutrient-dense options, such as smoothies, soups, yogurt-based products, and protein-enriched beverages, may improve dietary tolerance and facilitate adequate nutrient intake. Targeted supplementation, including vitamin D, calcium, and vitamin B12, should be guided by individual nutritional status and clinical need, with regular reassessment of dietary intake, hydration, and micronutrient adequacy throughout treatment [6,163,164,166]. Figure 2 summarizes the nutritional management strategies for several common adverse effects associated with incretin-based pharmacotherapy.

### 7.7. Leveraging Artificial Intelligence for Personalized Dietary Interventions

Recent advances in AI and ML have substantially expanded the potential of precision nutrition by enabling the integration and analysis of large, heterogeneous datasets encompassing genetic profiles, metabolomic signatures, gut microbiome composition, continuous glucose monitoring outputs, dietary intake records, and longitudinal lifestyle parameters. Unlike conventional nutritional approaches grounded in population-level dietary recommendations, AI-driven systems are capable of detecting significant interindividual variability in metabolic and postprandial responses to specific foods and dietary patterns, thereby facilitating the development of more personalized and adaptive nutritional interventions. Through predictive modeling and real-time data analysis, AI-based technologies can support individualized meal planning, optimization of glycemic control, prediction of pharmacological and dietary treatment response, and improvement of long-term dietary adherence. Emerging evidence further indicates that AI-assisted nutritional interventions contribute to clinically meaningful improvements in body weight management, metabolic health, and cardiometabolic risk profiles in individuals with obesity and related metabolic disorders [175,176]. Furthermore, AI-driven tools are increasingly being explored as adjuncts to incretin-based pharmacotherapy, offering the potential to tailor nutritional support to the specific metabolic and gastrointestinal challenges associated with GLP-1 and dual GIP/GLP-1 receptor agonist treatment [177,178]. Notably, AI is also becoming highly useful in anti-obesity drug discovery, unlocking next-generation therapeutics [179].

Likewise, AI technologies are being progressively integrated into mobile health applications, wearable monitoring devices, dietary assessment platforms, and virtual coaching systems that enable continuous physiological monitoring and individualized behavioral feedback. ML algorithms, image-recognition tools, natural language processing systems, and large language models are facilitating dietary tracking, automating nutritional assessment, supporting behavioral change strategies, and enhancing patient engagement throughout the treatment continuum. AI-based approaches also demonstrate considerable promise for identifying maladaptive eating behaviors, anticipating adherence challenges, and dynamically adjusting nutritional interventions in response to evolving physiological and behavioral patterns over time. Nevertheless, despite this considerable potential, important limitations remain. These include concerns regarding data privacy and security, algorithmic transparency and interpretability, equitable access across diverse socioeconomic and ethnic populations, and the limited external validation of AI models in real-world clinical settings. Addressing these challenges will require robust, large-scale, and longitudinal clinical studies, as well as the development of standardized regulatory frameworks for AI-driven nutritional tools, before their widespread implementation in routine obesity care can be responsibly recommended [180,181].

## 8. Current Limitations and Future Perspectives

While precision nutrition and omics-based strategies offer unprecedented opportunities for individualized obesity care, several significant limitations must be addressed before widespread clinical implementation can be achieved. First, the high costs associated with omics profiling (genomics, metabolomics, microbiome sequencing) and advanced continuous monitoring devices currently restrict their accessibility, often limiting these interventions to research settings or affluent populations [182,183]. This raises concerns regarding socioeconomic inequalities, as individuals most disproportionately affected by the obesity epidemic may have the least access to personalized care [184].

Furthermore, there is a distinct lack of standardized protocols for data collection, analysis, and interpretation across different healthcare systems. The integration of multi-omics datasets remains challenging from both computational and clinical points of view [185]. Furthermore, while several short-term studies demonstrate the potential of personalized nutrition, there is a scarcity of long-term randomized controlled trials validating the sustained clinical efficacy and cost-effectiveness of these approaches compared to standard care [186].

Looking forward, the successful translation of precision nutrition into routine practice will require the development of multidisciplinary obesity care models that integrate dietitians, geneticists, endocrinologists, and psychotherapists [182,187]. Integrating these tools into primary care will depend strongly on user-friendly digital health platforms and AI tools capable of synthesizing complex data into actionable advice for both practitioners and patients [185]. Finally, clear clinical guidelines, robust practitioner training, and stringent ethical frameworks regarding genetic privacy and AI-driven healthcare data will be essential to ensure that precision nutrition is delivered in a safe, equitable, and effective manner [188,189].

## 9. Conclusions

The management of obesity is undergoing a fundamental transformation, moving from generalized calorie-restriction models toward highly individualized, multi-dimensional strategies grounded in the biological heterogeneity of the disease. This review highlights that the evidentiary strength of precision nutrition varies across its pillars. Genetic profiling offers clear utility for monogenic obesity but limited translational benefit when used to match diet type to common polygenic variants, though genetic information may still improve outcomes when leveraged for risk disclosure and engagement rather than strict genotype-diet matching. Similarly, microbiome-targeted studies reveal that the *Bacillota* to *Bacteroidota* ratio shows inconsistent associations with obesity across studies, with genus-level and functional alterations offering more reproducible targets. Metabolomic approaches to personalized glycemic response have progressed from foundational predictive models toward large-scale clinical validation, while lipidomic pathway analyses offer additional, underutilized avenues for individualized guidance. Chrono-nutrition emerges as a promising but still underexplored pillar warranting further investigation.

The integration of personalized nutrition is also crucial in the era of advanced obesity pharmacotherapy. As incretin-based obesity medications become central to clinical care, tailored dietary strategies are essential to mitigate gastrointestinal adverse effects, ensure adequate micronutrient intake, and preserve lean body mass during weight loss. Looking forward, artificial intelligence and machine learning will be instrumental in translating complex multi-omics and lifestyle data into actionable, real-time nutritional guidance. Despite current challenges related to data privacy, algorithm validation, and equitable access, precision nutrition holds the potential to redefine obesity care, offering more effective, sustainable, and evidence-based, patient-centered therapeutic outcomes.

These conclusions, however, should be interpreted in light of the methodological approach adopted in the present review. The review is subject to several limitations inherent to its narrative design. First, as a non-systematic narrative review, it does not follow a pre-registered protocol or a PRISMA-based selection process, and consequently it is more susceptible to selection and citation bias than a systematic review or meta-analysis. Second, the literature search was restricted to publications available in English, which may have excluded relevant evidence published in other languages. Third, the findings were synthesized qualitatively, without quantitative pooling of effect sizes, meaning that the conclusions drawn should only be interpreted as a critical, integrative overview of the field rather than a precise, statistically weighted estimate of effect. These limitations should be considered when interpreting the conclusions of this review, and future systematic reviews and meta-analyses are warranted to quantitatively confirm the trends discussed herein.

## Figures and Tables

**Table 1:** Adult BMI classifications for individuals aged 20 years and above [20].

Category	BMI (kg/m^2^)
Underweight	<18.5
Normal weight	18.5–24.9
Overweight/Pre-obese	25–29.9
Obesity Class I	30–34.9
Obesity Class II	35–39.9
Obesity Class III	≥40

**Table 2:** Selected Genes Causing Monogenic Obesity and Their Established Roles in the Leptin–Melanocortin Pathway.

Gene	Pathway/Signaling Role	Functional Consequence When Mutated	Ref.
*LEP*	Adipocyte-derived hormone signaling energy stores to the hypothalamic leptin-melanocortin pathway	Biallelic loss-of-function variants cause congenital leptin deficiency, resulting in impaired hypothalamic leptin signaling, severe hyperphagia, and early-onset obesity	[52]
*LEPR*	Leptin receptor in hypothalamic circuits; the first receptor-mediated step in leptin–melanocortin signaling	Biallelic loss-of-function variants abolish leptin-receptor signaling despite normal or elevated circulating leptin, causing hyperphagic early-onset obesity resembling congenital leptin deficiency	[53,54]
*POMC*	Precursor of α-MSH, the endogenous MC4R agonist; precursor of ACTH	Biallelic loss-of-function variants impair the production of POMC-derived peptides, causing severe early-onset hyperphagia and obesity, central adrenal insufficiency, and variably altered pigmentation	[54]
*PCSK1*	Prohormone convertase (PC1/3) that processes POMC and multiple neuroendocrine and enteroendocrine prohormones	Biallelic loss-of-function variants impair prohormone processing and downstream melanocortin signaling, causing severe early-onset obesity; severe neonatal or infantile malabsorptive diarrhea and multiple endocrinopathies are also characteristic	[55]
*MC4R*	Central, particularly hypothalamic, receptor mediating anorexigenic melanocortin signaling induced by α-MSH	Loss-of-function variants, frequently in the heterozygous state, represent the most common cause of non-syndromic monogenic obesity and disrupt the central control of food intake and satiety	[54]

Unlike the common polygenic variants summarized in Table 3, which were identified through statistical association in GWAS, the causal role and molecular function of the genes listed in this table were established through direct pedigree, biochemical, and functional studies, independent of GWAS. ACTH, adrenocorticotropic hormone; α-MSH, α-melanocyte-stimulating hormone; LEP, leptin; LEPR, leptin receptor; MC4R, melanocortin 4 receptor; PCSK1, proprotein convertase subtilisin/kexin type 1; POMC, proopiomelanocortin.

**Table 3:** Examples of Common, GWAS-Associated SNPs Statistically Linked to Obesity Susceptibility (Polygenic Risk Variants).

Gene	SNP	Chromosome Locus	Risk Allele	Functional Mechanisms	References
*FTO*	rs9939609	16q12.2	A	The most robustly associated obesity locus. The risk allele alters energy expenditure and increases preference for high-fat, energy-dense foods, leading to increased BMI and adiposity.	[64,71,72]
*MC4R*	rs17782313	18q21.32	C	Impairs the melanocortin signaling pathway in the hypothalamus, disrupting satiety signals. Strongly associated with increased energy intake, severe early-onset obesity, and hyperphagia.	[65,71,73]
*LEP*	rs7799039	7q32.1	A	Alters leptin gene transcription and secretion from adipose tissue. The risk allele is associated with altered energy homeostasis, increased BMI, and visceral fat accumulation.	[66,74]
*BDNF*	rs6265	11p14.1	A	Impairs BDNF secretion in the brain, negatively impacting the regulation of appetitive behavior and energy balance. Associated with increased food intake and BMI.	[68,75,76]
*ADIPOQ*	rs2241766/rs1501299	3q27.3	G/T	Reduces circulating adiponectin levels, impairing glucose and lipid metabolism. Associated with increased risk of central obesity and metabolic syndrome.	[77,78]
*PPARG*	rs1801282	3p25.2	C (Pro)	The Pro allele (wild-type) is associated with lower insulin sensitivity compared to the Ala allele. Interacts with dietary fat intake to influence BMI and obesity risk.	[67,79]
*FABP2*	rs1799883	4q28–q31	A	Increases intestinal fatty acid–binding affinity and dietary fat absorption. Associated with insulin resistance and elevated risk of obesity.	[69]
*APOA2*	rs5082	1q23	C	Reduces APOA2 promoter activity and expression. The risk allele interacts with high saturated fat intake to increase appetite and BMI, specifically among high-fat consumers.	[70]
*ADRB2*	rs1042714	5q32	C	Alters β2-adrenergic receptor function, reducing catecholamine-mediated lipolysis and energy expenditure. Associated with increased susceptibility to obesity.	[69]

Common susceptibility SNPs listed in this table should not be conflated with the rare, high-penetrance pathogenic variants associated with monogenic obesity. *MC4R* and *LEP* harbor rare pathogenic mutations causing monogenic obesity; the variants listed here instead reflect common polymorphisms conferring modest, population-level risk. Similarly, pathogenic *PPARG* mutations cause a distinct monogenic disorder, familial partial lipodystrophy type 3, marked by abnormal fat redistribution and severe insulin resistance rather than obesity per se; the Pro12Ala variant listed here is a separate, common polymorphism [80]. Large deletions spanning the *BDNF* locus (11p14) similarly cause obesity as part of WAGR syndrome, distinct from the modest effect of the common rs6265 variant discussed above [81]. ADIPOQ, adiponectin; ADRB2, adrenoceptor beta 2; Ala, alanine; APOA2, apolipoprotein A-II; BDNF, brain-derived neurotrophic factor; BMI, body mass index; FABP2, fatty acid-binding protein 2; FTO, fat mass and obesity-associated; GWAS, genome-wide association studies; LEP, leptin; MC4R, melanocortin 4 receptor; PPARG, peroxisome proliferator-activated receptor gamma; Pro, proline; SNPs, single nucleotide polymorphisms.

## Data Availability

No new data were generated or analyzed in this study. Data sharing is not applicable to this article.

## Abbreviations

The following abbreviations are used in this manuscript:

ACTH	adrenocorticotropic hormone
ActRII	activin type II receptors
ADA	American Diabetes Association
ADRB2	adrenoceptor beta 2
AgRP	Agouti-Related Peptide
α-MSH	α-Melanocyte-Stimulating Hormone
APOA2	apolipoprotein A-II
ARC	Arcuate Nucleus
BAT	Brown Adipose Tissue
BDNF	Brain-Derived Neurotrophic Factor
BeAT	Beige Adipose Tissue
BMI	Body Mass Index
CART	Cocaine- and Amphetamine-Regulated Transcript
CCK	Cholecystokinin
DEXA	Dual-Energy X-Ray Absorptiometry
DHA	Docosahexaenoic Acid
EASO	European Association for the Study of Obesity
EPA	Eicosapentaenoic Acid
FABP2	fatty acid-binding protein 2
Fiaf	Fasting-Induced Adipose Factor
FMT	Fecal Microbiota Transplantation
FOS	Fructo-Oligosaccharides
FTO	Fat Mass and Obesity-Associated Gene
GIP	Glucose-dependent Insulinotropic Polypeptide
GLP-1	Glucagon-Like Peptide-1
GLP-1 RAs	Glucagon-Like Peptide-1 Receptor Agonists
GOS	Galacto-Oligosaccharides
GWAS	Genome-Wide Association Studies
LEDs	Low-Energy Diets
LEPR	Leptin Receptor
MHO	Metabolically Healthy Obesity
ML	Machine Learning
MUFAs	Monounsaturated Fatty Acids
MUO	Metabolically Unhealthy Obesity
NCDs	Non-Communicable Diseases
NPY	Neuropeptide Y
PCSK1	Proprotein Convertase Subtilisin/Kexin Type 1
POMC	Proopiomelanocortin
PPARG	Peroxisome Proliferator-Activated Receptor Gamma Gene
PPG	Postprandial Glucose
PPGRs	Postprandial Glycemic Responses
PUFAs	Polyunsaturated Fatty Acids
PYY	Peptide YY
SCFAs	Short-Chain Fatty Acids
SFAs	Saturated Fatty Acids
SNPs	Single Nucleotide Polymorphisms
TRE	Time-Restricted Eating
UCP1	Uncoupling protein 1
VLEDs	Very-Low-Energy Diets
WAT	White Adipose Tissue
WHO	World Health Organization
